# Non-Fistulous Abscess

**Published:** 1886-03

**Authors:** 


ARTICLE II.

### NON-FISTULOUS ABSCESS.

BY DR. KULP.

I understand the subject is diagnosis and treatment of a dormant abscess, or an abscess that has not a fistulous opening, *i. e.*, is not giving any perceptible trouble. Of course, when we have pain and swelling we may expect the formation of pus, and later an abscess. I think that many of these cases give a very great degree of trouble, because we over-treat them.

The question has been asked, why is it after the cavity has been opened we have pain ? I have yet to know when there was no pain previously that immediately when I opened the cavity I caused pain unless I take an instrument and force it up into the canal, or force something through the opening, either air or something else. In that class of uncertain cases I use a great deal of care, for even forcing up a broach will force up air to disturb it. It is necessary to be exceedingly cautious. If I find the nerve is obliterated, if I find a lump of enlargement on the outside, one of these old chronic cases, I should open the canal cautiously. After opening it, I direct the patient to perform the operation of suction upon the tooth. Then place a little piece of cotton saturated with creosote and tannin and place it in the root. I am cautious not to place it so firmly, as to force up air. Let it remain for twenty-four hours, then place in dry cotton, which will absorb all the exudation of pus that may come from the end of root. If you thus encourage nature instead of disturbing her, you will seldom be troubled with the pain complained of. This treatment can be continued until there is no further formation of pus; then you can fill the tooth. If I should have trouble after the tooth was filled I should take Dr. Ingersoll's plan of stimulating and assisting nature. Instead of capsicum I should take a saturated solution of creosote and iodine. As to the point of difference between abscess and ulceration of the peridental membrane, I agree with him that we may have pus at the end of the root without an abscess. I do not believe we have an abscess where there is no fistulous opening; you will find that the greatest trouble comes from teeth having an abscess and drainage through the canal; when you come to close up the drainage you will have trouble; it may become necessary to force a fistulous opening before you succeed in those cases. In cases of this kind I have found my greatest success to be with the use of creosote and tannin. Dip cotton in creosote and then in dry tannin, then place it in the canal very carefully so as not to force anything through the root. In twenty-four hours remove it and then dress with dry cotton; that will usually stop the trouble. There is trouble in over-treating. As soon as the pus secretion ceases, while nature is making an effort to carry away this membrane forming at the root of the tooth, at once fill the tooth. By continuing to treat it from day to day and from week to week, you will never get your case well. I have filled for years all canals, particularly canals of this kind with os-artificial.

The method I adopt is to put a little powder on a glass slab, and then some of the liquid by the side of it; then take a smooth pointed broach and put on it a shred of cotton, wrapping it smoothly, and then with that instrument and the cotton on it, the powder is pushed into the liquid and mixed, making it about as thick as cream. The quantity on the cotton is sufficient to fill the canal. Put the broach up into the root as far as you can; then, by giving it a pumping motion, you can withdraw the broach from the cotton, with the cotton drawn back into the larger part of the canal; take hold of the end of the cotton and pump it up until you have exhausted all the air out of the root, np to the apex. Your patient will flinch just as you reach the apex. The great point of caution I want to make is the danger of over-treatment of these cases. I don't think they are so very difficult, if proper care is taken in the first steps. I do not see any reason why we should make so much ado about such cases, when we create the mischief ourselves. There are some cases where there is thickening of the peridental membrane. That is not blind abscess. I call that ulceration of the peridental membrane, and treat that as I would an ulcer. I do not think the profession know fully the value of tannin combined with carbolic acid and creosote.—*Southern Dental Journal.*

---

ARTICLE III.

